# Supplementary figures and images for: The efficacy of Topical Clascoterone versus systematic spironolactone for treatment of acne vulgaris: A systematic review and network meta-analysis
Source: PLoS One. 2024 May 30;19(5):e0298155. doi: 10.1371/journal.pone.0298155 (PMC11139337; doi:10.1371/journal.pone.0298155)

## Slide 1
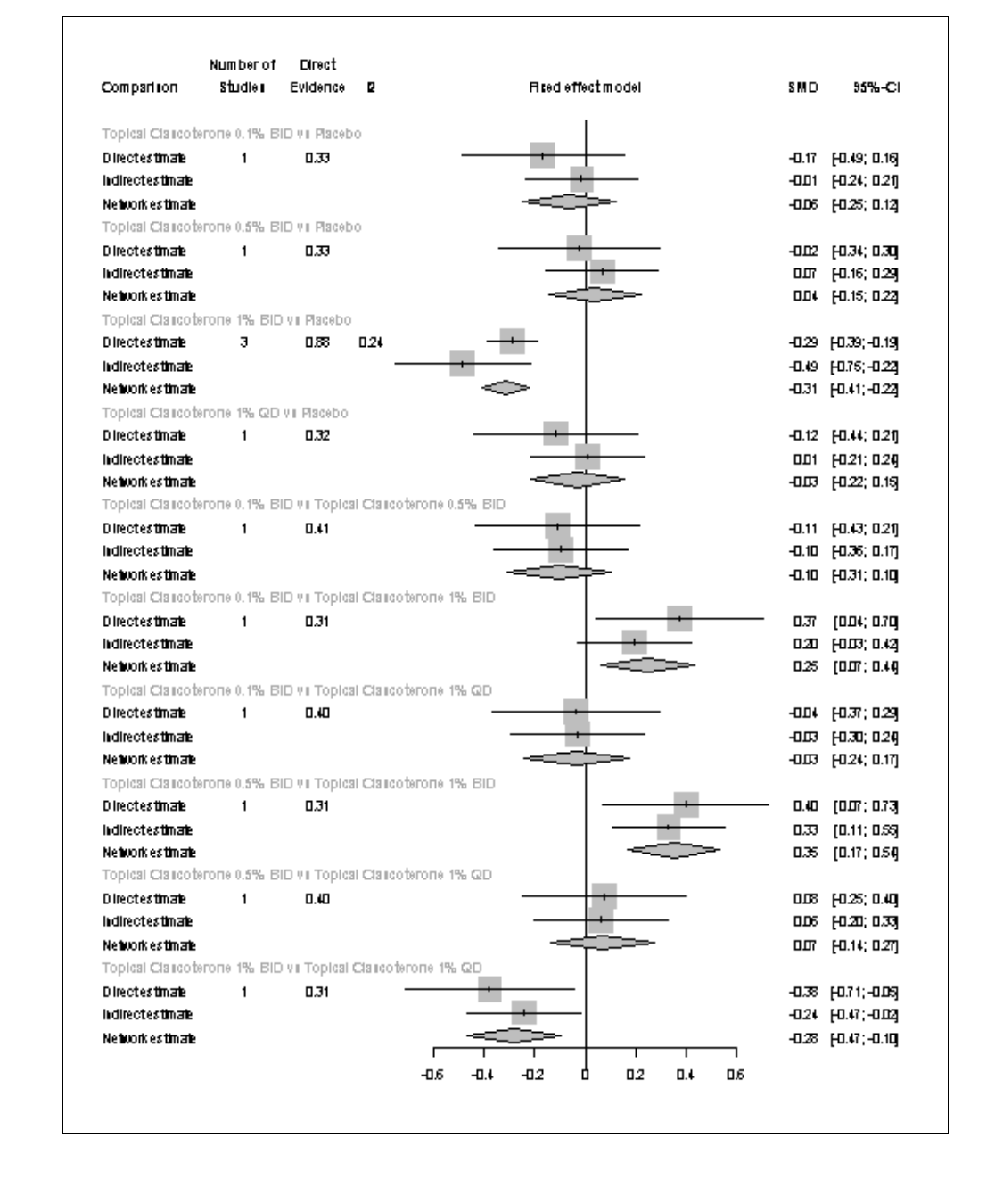

## Slide 2
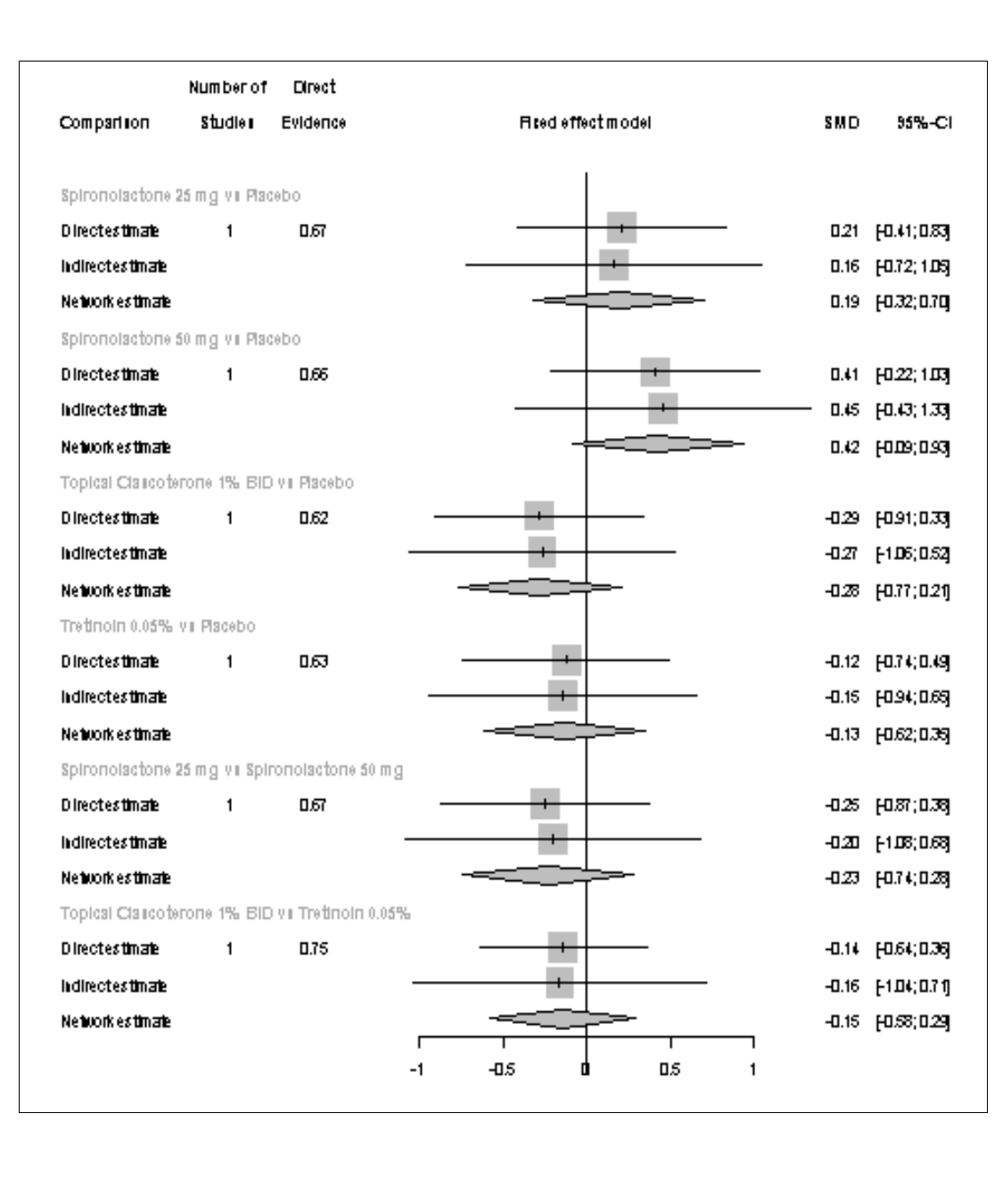

## Slide 3
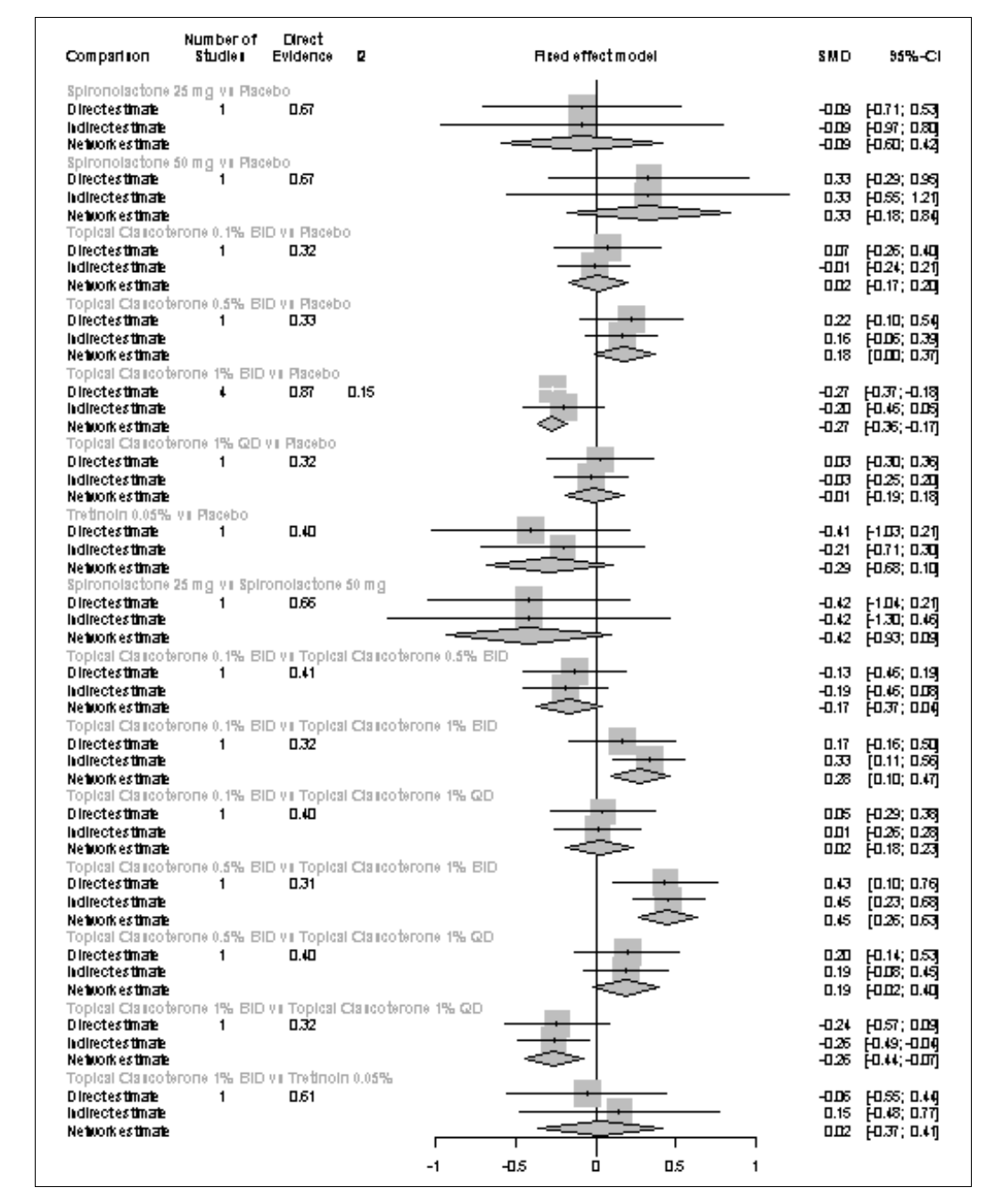

## Slide 4
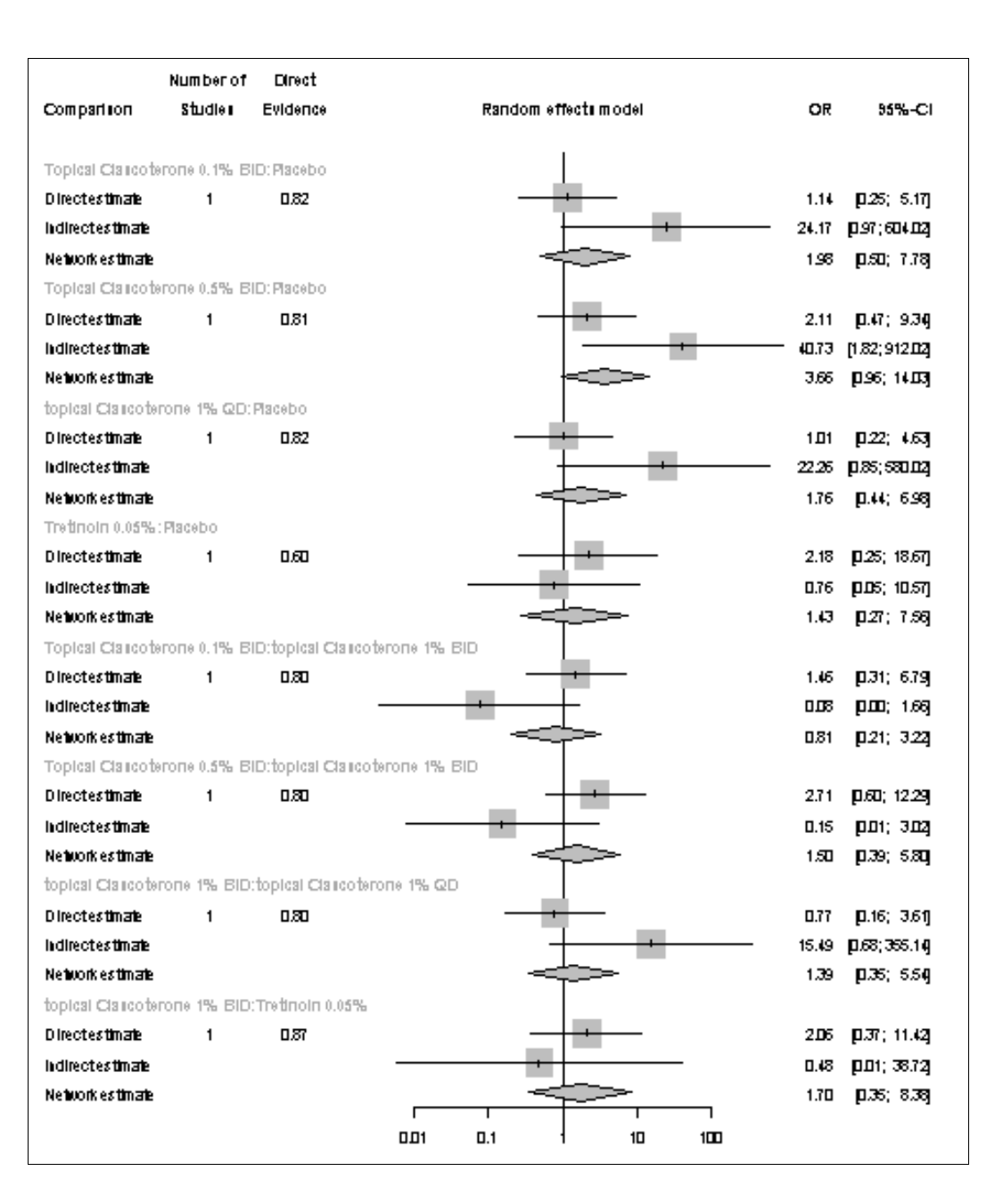

Supplement: S1 File — (PPTX) [file pone.0298155.s002.pptx]
