# Supplementary material for: The efficacy of Topical Clascoterone versus systematic spironolactone for treatment of acne vulgaris: A systematic review and network meta-analysis
Source: PLoS One. 2024 May 30;19(5):e0298155. doi: 10.1371/journal.pone.0298155 (PMC11139337; doi:10.1371/journal.pone.0298155)
Supplement: Attachment — Submitted filename: Meta Analysis.docx [file pone.0298155.s003.docx]

**Reviewer(s) & Comments to Author:**

The Systematic review article entitled “The Efficacy of Topical Clascoterone versus Systematic Spironolactone for Treatment of Acne Vulgaris: A Systematic Review and Network Meta-Analysis”. In this article authors have done meta-analysis computerized search through PubMed/MEDLINE, SCOPUS, and the Cochrane Library and this work sheds light on acne vulgaris treatment in clinical settings. The work seems to interesting in reading, a succinct account of the issues and concerns is as follows.

1. In introduction section authors should add the statistical estimate for cases on Acne Vulgaris per year basis.

2. What is the reason for comparing two different classes of drug based on their pharmacological effect? Spironolactone approved for used as diuretic and Clascoterone act as anti-androgen in treatment of acne. Also their route of administration is also different.

3. The title of table 2. “Direct and pooled comparisons for NILC” suggested to modify it to “Direct and pooled comparisons for NILC of Clascoterone at different dose”

4. Table 1. Lacks data of random clinical trials on male subjects.

5. Why Tretinoin comparison data for IGS is added in manuscript, when study is focused on Clascoterone and spironolactone

6. Whether the study was registered on PROSPERO or not.

7. What keywords were used and how many total search results were found for this study.
