# Supplementary material for: The efficacy of Topical Clascoterone versus systematic spironolactone for treatment of acne vulgaris: A systematic review and network meta-analysis
Source: PLoS One. 2024 May 30;19(5):e0298155. doi: 10.1371/journal.pone.0298155 (PMC11139337; doi:10.1371/journal.pone.0298155)
Supplement: Attachment — Submitted filename: Point-to-point response.docx [file pone.0298155.s004.docx]

Dear Editor,

We would like to thank you for your time and efforts. Please find the point-to-point response:

| Reviewer | Comment | Response |
| --- | --- | --- |
| Reviewer #3 | The methodology is still not expalined in an explicit fashion; even the route of administration is not defined. Removing the bullets does not solve the purpose, it should be written in complete sentences making a paragraph. | Thank you for your comment. We have added the route of administration, it is topical Clascoterone or  Tretinoin and oral Spironolactone. The eligibility criteria were rewritten to be as one paragraph |
| Reviewer #4: | I come to this paper as a new reviewer on the first revision.  Please do not use the term pooled analysis - meta-analysis is quite different from pooled analysis as there is only comparison within trial and then estimates are combined - a pooled analysis does not do this. | Thank you for your comment. We have addressed this point and removed “Pooled analysis”. |
|  | Why are both fixed (assumption-free) and random-effects methods used - the two have very different underlying approaches. Had the Q statistic been higher in the fixed case would a random-effects model have been used - in which case a random effects model was the method of analysis and the results are those for a random effects analysis with low Q. | Thank you for your comment, based on the Cochrane Handbook instructions, we used the fixed-effects (plural) model by default to calculate weighted SMDs and 95% Confidence Intervals (CI) in NMA. In cases of heterogeneity among the included studies, a random-effects model was employed. The I2 and Tau2 statistics were utilized to express the inter-trial heterogeneity. |
|  | In a network meta-analysis one issue is that one needs to know the patients are comparable and not contraindicated for one or other treatment. Otherwise in a cancer scenario one may combine fit and unfit patients and conclude erroneously that intensive treatment be given to unfit patients. Please explain how this was ensured here. | Dear Reviewer, we would like to thank you for highlighting this, we confirm that the baseline characteristics of all patients across the included studies did not show that any of these regimens are contraindicated in the other group. |
|  | Given the doses here can one model the dose response relationship? Confidence intervals are wide here and it is incorrect to dichotomise significance as one cannot rule out meaningful differences and the 1% qd result seems out of line with the bid result. Given that qd looks like qid maybe od is a better abbreviation here for clarity. | Thank you for your comment. We have added a new section “3.8. Dose-response”, based on your suggestions |
|  | Is information given in the publication on prior lines of therapy? | Yes, please refer to the third paragraph on the introduction section. |
