# Supplementary material for: The efficacy of Topical Clascoterone versus systematic spironolactone for treatment of acne vulgaris: A systematic review and network meta-analysis
Source: PLoS One. 2024 May 30;19(5):e0298155. doi: 10.1371/journal.pone.0298155 (PMC11139337; doi:10.1371/journal.pone.0298155)
Supplement: Attachment — Submitted filename: Point-to-point response (1).docx [file pone.0298155.s005.docx]

Dear Editor,

We would like to thank you for your time and efforts. Please find the point-to-point response:

| Reviewer | Comment | Response |
| --- | --- | --- |
| Reviewer #3 | No Response | Thank you for your feedback. |
| Reviewer #4: | Thank you for your response to my previous comments. I have a few remaining comments  1 The use of the word "pooled". There are still instances here, e. Section 3.4 line 4 (where the word should be excised- the data did not show heterogeneity" - the test here does not show homogeneity but rather shows no evidence of heterogeneity - these are two different concepts) - the same thing is in sections 3.5 and 3.6; Legend to Table 2, 3, 4, 5. I think in all these cases the "pooled" analysis results are the network meta-analytic estimates? | Thank you for your comment. We have addressed this point and removed “Pooled analysis”. In addition, we replaced “the data did not show heterogeneity” with “shows no evidence of heterogeneity” as recommended. |
|  | 2. In terms of type of meta-analysis used then you are using random effects throughout as this reduces to fixed effects if Q<= number of degrees of freedom because of dividing by zero or negative numbers- it would make it more consistent if this was stated as otherwise it looks like you are picking and choosing. | Thank you for your comment, based on the Cochrane Handbook instructions, we used the fixed-effects (plural) model by default to calculate weighted SMDs and 95% Confidence Intervals (CI) in NMA. In cases of I^2^ is >50% and p-value is <0.10, we use random model. |
|  | 3. This response has not made it into the paper as I can see - please add this explanation to demonstrate mathodological validity. | Dear Reviewer, we would like to thank you for highlighting this, we added this point to the section on characteristics of included studies. |
|  | 4. QD remains a potentially misleading abbreviation as it could be read as qid. I would still request the use of ODS for once daily | Thank you for your comment. We have replaced QD with ODS. |
|  | 5. For dose response we need evidence of heterogeneity between doses instead of dichotomising significance. Please add this | Dear Reviewer,  We are not sure if we understand your comment correctly; however, we included the results of heterogeneity for the dose-response, if this is what you asking for. If not, please elaborate more about your request. |
